# A Critical Exploration of the Total Flavonoid Content Assay for Honey

**DOI:** 10.3390/mps7060095

**Published:** 2024-11-21

**Authors:** Sharmin Sultana, Ivan Lozada Lawag, Lee Yong Lim, Kevin J. Foster, Cornelia Locher

**Affiliations:** 1 Division of Pharmacy, School of Allied Health, University of Western Australia, Perth 6009, Australia; sharmin.sultana@research.uwa.edu.au (S.S.); ivan.lawag@uwa.edu.au (I.L.L.); lee.lim@uwa.edu.au (L.Y.L.); 2 Institute for Pediatric Perioperative Excellence, The University of Western Australia, Perth 6009, Australia; 3 Institute of Herbal Medicine, National Institutes of Health, University of the Philippines Manila, 1st Flr., Paz Mendoza Building, UP College of Medicine, 547 Pedro Gil St., Ermita, Manila 1000, Philippines; 4 School of Agriculture and Environment, University of Western Australia, Crawley 6009, Australia; kevin.foster@uwa.edu.au; 5 Department of Primary Industries and Regional Development, Perth 6000, Australia

**Keywords:** total flavonoid content (TFC) assay, AlCl_3_, honey, UV–Vis spectrophotometry, blanking

## Abstract

This study critically investigates the aluminium chloride–based colorimetric determination of the total flavonoid content (TFC) of honey. Following a comprehensive review of the recent literature reporting the use of the assay in the determination of TFC in honey, 10 honeys of different botanical origins were investigated using the colorimetric method alongside an artificial honey that was used as a control. Using spiking experiments, this study demonstrates that the flavonoid concentrations commonly found in honey are too low for a direct measurement and thus some of the TFC data reported in the literature might more likely be a reflection of the honey’s inherent colour rather than a product of the coordination complex formed specifically between flavonoids and Al^3+^ ions. This paper highlights the importance of correct blanking and suggests alternative approaches to the traditional TFC assay for honey to ensure analysis results that are truly reflective of honey’s TFC.

## 1. Introduction

Honey, a supersaturated sugar solution, is not only a popular food and flavouring agent, but also a commonly used natural remedy. Its use as complementary medicine stems primarily from its antibacterial and antioxidant activities [1,2]. Honey is mainly produced from the nectar of flowers, which bees collect and convert into honey with the help of bee-derived enzymes [3]. Honey contains 80–85% carbohydrates (mainly fructose and glucose), 15–17% water, approximately 0.3% protein, and about 0.2% minerals. Furthermore, amino acids, organic acids, phenolics such as flavonoids, and vitamins are also present at low levels, together making up about 3% of the honey’s total weight [1,2,4,5,6].

Many of the therapeutic effects ascribed to honey, such as its antioxidant and anti-inflammatory properties [7,8,9,10,11], are mainly related to its polyphenol profile, which captures heterogeneous classes of compounds that can be categorised into flavonoids and phenolic acids [8,9,10,11,12,13]. They are secondary metabolites of plants and transferred from the flower nectar into honey by bee activity. Thus, the amount and type of polyphenols present in honey mainly depend on its botanical source. However, geographical factors might also come into play as ecological and climatic features, such as weather conditions, soil type, rainfall, or soil mineral content, also influence the nectar’s chemical composition [14,15]. Thus, honeys derived from the same botanical source but from different geographical regions may differ in their chemical composition, including their flavonoid profile, and with this also their levels of bioactivity.

Flavonoids are an important class of natural products. They serve as flower pigments to attract pollinators in most Angiosperm families, but their occurrence is not restricted to flowers as they are found in all parts of plants where they promote growth and are involved in various defence mechanisms [16,17,18]. They are also associated with a broad spectrum of health-promoting effects due to their antioxidative, anti-inflammatory, anti-mutagenic, and anti-carcinogenic properties due to their capacity to interact with key cellular enzymes such as xanthine oxidase (XO), cyclo-oxygenase (COX), lipoxygenase, and phosphoinositide 3-kinase [17,18,19,20]. Subsequently, there is a strong interest of consumers in plant extracts and food items that are rich in flavonoids.

**Figure 1:**
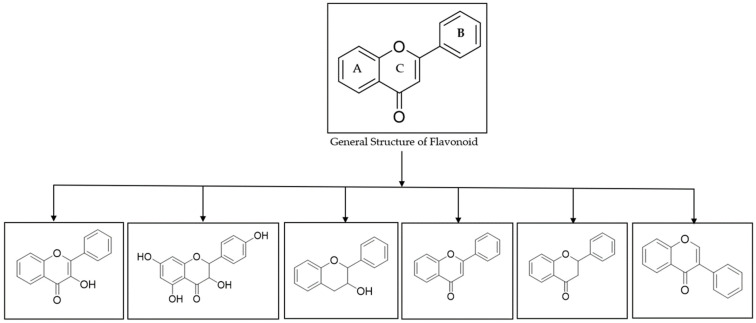
General structure and subclasses of flavonoids [21].

Chemically, flavonoids can be divided into different subgroups (Figure 1) [21], comprising flavonols, flavononols, flavan-3-ols, flavones, flavonones, and isoflavones, depending on which carbon of the C ring the B ring is attached to and the molecules’ substitution, degree of saturation and oxidation [16,22,23].

Flavonoids are frequently detected components in honey and have been linked to its antioxidant, anti-inflammatory, and antimicrobial effects [16,17,18,19,20,22,23]. Consequently, there is strong interest in the continued identification and quantification of flavonoids in various honeys harvested around the world.

**Figure 2:**
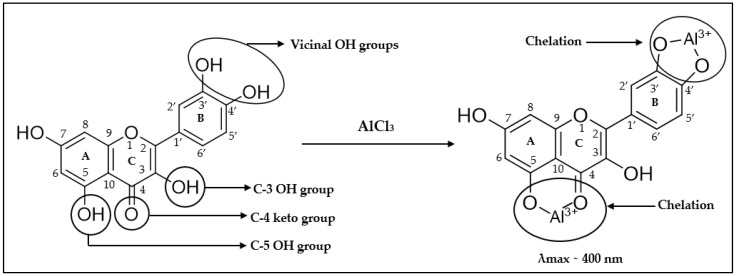
Formation of an Al (III)-flavonoid chelate [21].

Typically, total flavonoid content (TFC) is used to capture the entirety of flavonoids present in honey and other natural products. TFC is also employed as a quality parameter with the assumption that a higher TFC is associated with stronger antioxidant and thus health-beneficial activities. A colorimetric assay using aluminium chloride (AlCl_3_) was first proposed by Christ and Müller in 1960 for the determination of the content of flavonol derivatives in drugs [24] and the approach has since been frequently used to determine the TFC in honey. The traditional assay (which has undergone several modifications, for example, the addition of NaNO_2_ and NaOH to the reagent to enhance the sample response or the addition of KC_2_H_3_O_2_ to AlCl_3_ or using Al(NO_3_)_3_ with KC_2_H_3_O_2_. These modifications are, however, outside the scope of this study, and the traditional assay, using only AlCl_3_ as a reagent, is referred in this paper simply as ‘colorimetric assay’ or ‘TFC assay’) is based on the formation of a coordination complex involving the Al^3+^ cation, either as an acid-stable complex involving the flavonol’s C-4 keto group and its C-3 or C-5 hydroxyl group, or an acid-labile complex based on vicinal dihydroxyl groups in the B-ring of flavonoids (Figure 2) [21]. The absorbance maximum of the Al (III)-flavonoid chelates is around 400 nm. The TFC of a sample is then expressed as quercetin equivalent per gram of the investigated sample using a standard curve prepared from various concentrations of the reference flavonoid [25].

Though widely popular, the traditional AlCl_3_ colorimetric assay for the determination of TFC has several inherent flaws, such as high false-positive or false-negative results drugs [24]. Moreover, the method does not identify the types of flavonoids present, and it is also unsuitable for the determination of certain flavonoid subtypes, such as isoflavones where specific ring substitutions do not allow for complexation with Al^3+^ [25]. Additionally, by virtue of it being a simple colorimetric method, the TFC assay does not immediately allow for differentiation between sample constituents that naturally have absorption maxima of about 400 nm even without complexing with AlCl_3_ and the flavonoids that produce an absorbance reading at 400 nm only after complexation with AlCl_3_. This potential limitation is of particular relevance to the determination of TFC in honey as the typical yellow, golden or brown colouration of honeys can be expected to produce a natural absorbance around 400 nm that could potentially interfere with the AlCl_3_ colorimetric assay. In the light of these challenges, a careful consideration of a suitable blanking solution is warranted if the AlCl_3_ colorimetric assay is used for TFC determination in honey.

The objectives of the present study were firstly to conduct a comprehensive review of the literature to gauge the popularity of TFC determination in honey and to document commonly adopted assay conditions. This was followed by a critical exploration of the TFC assay with a particular focus on the use of a suitable blanking solution, and the impact of blanking on the assay results. Based on the findings of this investigation, alternative approaches to the traditional TFC assay for honey are suggested.

## 2. Literature Review

A review of the literature published over the past four years (2021–2024) was conducted using the Scopus database and the search terms ‘honey’ and ‘total flavon’ to determine the current frequency of use of the AlCl₃ method as an analytical tool for TFC determination in honey. A total of 54 research publications were retrieved that had reported the use of this assay for the determination of TFC in honeys (Table 1). The honey samples are well described and comprise a wide range of monofloral and multifloral honeys harvested from different regions in the world (e.g., Asia, Africa, Europe). The assay methodologies show several similarities (Table 1), for example detection wavelengths employed are within the narrow range of 405 to 437 nm. Quercetin emerged as the preferred standard (45 out of 54) for quantifying the flavonoid equivalence in the honey samples facilitating comparisons of flavonoid levels across different honey types. However, of the 54 reviewed papers, only 11 stipulate the specific blanking solution used in the assay. This lack of detail in many of the published assay methodologies served as the impetus for this study, which was to explore and validate assay conditions that allow for a reliable determination of the TFC of honey, while minimizing overestimation caused by the honey’s inherent colour.

## 3. Materials and Methods

### 3.1. Chemicals and Reagents

All reagents and solvents were of analytical grade. Quercetin was obtained from ChemFaces (Wuhan, China), and methanol was purchased from Scharlau (Barcelona, Spain). Anhydrous aluminium chloride was obtained from Sigma-Aldrich (Darmstadt, Germany). Aluminium chloride solution (10% *w*/*v*) was prepared by dissolving 10 g of the reagent in methanol and making the volume up to 100 mL.

### 3.2. Honey Samples and Organic Honey Extracts

This study used a range of honeys of different floral origins (Table 2) alongside an artificial honey. The artificial honey was prepared by dissolving 1.5 g sucrose, 7.5 g maltose, 40.5 g fructose, and 33.5 g glucose in 17 mL of deionised water [77]. All honey samples, including the artificial honey, were prepared for analysis as follows:

(1) 20% (*w*/*v*) aqueous solutions.
(2) 20% (*w*/*v*) aqueous solutions spiked with quercetin to serve as positive controls. For this, a 0.05% (*w*/*v*) quercetin solution in methanol was prepared. Each honey sample (0.4 g) was spiked with 140 µL of the quercetin solution (70 µg of quercetin) before being dissolved in and made up to 2 mL of deionised water.
(3) Honey extracts were also prepared by dissolving 5 g of each honey in 10 mL of deionised water, followed by three extractions with 5 mL of acetonitrile and dichloromethane (1:1, *v*/*v*). The combined organic extracts were dried with anhydrous MgSO_4_, filtered, and the solvent evaporated under a nitrogen stream before being reconstituted in 2 mL of water to yield aqueous honey extracts. The same extraction method was also used for the artificial honey spiked with 70 µg quercetin.

### 3.3. Quercetin Calibration Curve

A stock solution of 0.05% (*w*/*v*) quercetin was prepared by dissolving 5 mg of quercetin in 10 mL of methanol. Using different blanking approaches, two 5-point standard curves were prepared using 20, 40, 60, 80, and 100 µL of the stock solution made up to 2 mL with deionised water.

### 3.4. Colorimetric Assay

To 2 mL of each of the 20% (*w*/*v*) aqueous honey samples, 2 mL of 10% AlCl₃ solution was added and the resulting absorbance was measured after 30 min at 400 nm. To investigate the impact of blanking on the absorbance reading, two types of blanking solutions were used: (a) a mixture of 2 mL of water and 2 mL of methanol and (b) 2 mL of the respective aqueous honey solution mixed with 2 mL of methanol (Table 3).

To 2 mL of all quercetin-spiked honey solutions, 2 mL of 10% AlCl_3_ solution was added, and the resulting absorbance was measured after 30 min at 400 nm. In this assay, two types of blanking solutions were also used: (a) a mixture of 2 mL of water and 2 mL of methanol and (b) 2 mL of the respective spiked aqueous honey solution mixed with 2 mL of methanol (Table 4 and Table 5).

To 2 mL of all honey extracts and the quercetin-spiked artificial honey extract, 2 mL of 10% AlCl_3_ solution was added, and the resulting absorbance was measured after 30 min at 400 nm after blanking with 2 mL of the respective quercetin-spiked aqueous honey extract mixed with 2 mL of methanol (Table 6).

**Figure 3:**
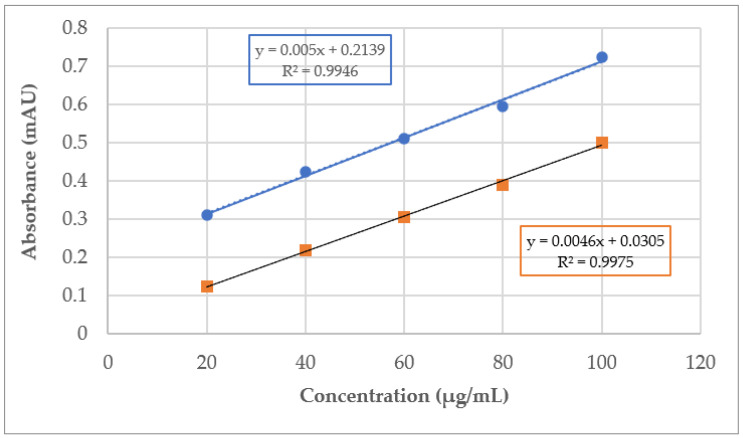
Quercetin calibration curves: orange line—blanked with methanolic quercetin solution (calibration curve 1) and blue line—blanking with methanol (calibration curve 2).

To 2 mL of the different concentrations of quercetin standards, 2 mL of 10% AlCl_3_ solution was added, and the resulting absorbance was measured after 30 min at 400 nm to prepare the quercetin calibration curve. To investigate the impact of blanking, two types of blanking solutions were used: (a) 2 mL of methanol and (b) 2 mL of methanolic quercetin solution (Figure 3).

## 4. Results

To investigate the impact of blanking, the assay was first carried out with the various honey samples and also the artificial honey using either a water–methanol solution or an aqueous honey–methanol solution for blanking. The results of this investigation are summarised in Table 3.

**Table 3:** Impact of blanking on absorbance readings of 20% aqueous honey solutions.

Honey	Absorbance at 400 nm	
	Blanking with Water–Methanol Solution	Blanking with Aqueous Honey–Methanol Solution
Red Clover Honey	0.378	0.067
Sainfoin Clover Honey	0.365	0.061
Manuka Honey	0.747	0.174
Jarrah Honey	0.751	0.176
Marri Honey	0.403	0.071
Peppermint Honey	0.795	0.161
Blackbutt Honey	0.608	0.093
Melaleuca Honey	0.419	0.091
Watermelon Honey	0.777	0.172
Bush Honey	0.778	0.177
Artificial Honey	0	0

To increase the flavonoid concentration and thus ensure that absorbance readings were within the Beer–Lambert range (absorbance between 0.3 and 0.8), the above experiment was repeated with honeys spiked with quercetin, again using two different blanking solutions (Table 4).

**Table 4:** Absorbance readings of different honeys spiked with quercetin after blanking with a spiked aqueous honey–methanol or a water–methanol solution.

Honey	Absorbance at 400 nm	
	Blanking with Quercetin-Spiked Aqueous Honey–Methanol Solution	Blanking with Water–Methanol Solution
Red Clover Honey	0.41	0.988
Sainfoin Clover Honey	0.404	0.969
Manuka Honey	0.517	1.464
Jarrah Honey	0.517	1.468
Marri Honey	0.414	1.017
Peppermint Honey	0.504	1.499
Blackbutt Honey	0.436	1.244
Melaleuca Honey	0.434	1.053
Watermelon Honey	0.515	1.492
Bush Honey	0.520	1.497
Artificial Honey	0.343	0.652

Figure 3 shows two 5-point calibration curves (calibration curve 1 and 2) prepared using two types of blanking solution as described in Section 3.3 and Section 3.4.

A quantitative assay was also carried out using the artificial honey sample spiked with a known amount of quercetin as described in Section 3.2, using either quercetin-spiked aqueous honey–methanol solution or a water–methanol solution for blanking (Section 3.4). In the former case (using calibration curve 1), 98.3% of the theoretical amount of quercetin was detected in the spiked sample, whereas blanking with methanol (using calibration curve 2) resulted in a significant (124.84%) overestimation of quercetin content in the spiked artificial honey sample. Table 5 presents the TFC content of the flavonoid-spiked honeys expressed as quercetin equivalents (QE) per gram of the sample, which is a quantification approach commonly used in TFC determination, using calibration curve 1. For this, the actual amount of total flavonoid content present in these natural honeys was determined by subtracting the determined TFC of the spiked artificial honey (69.18 µg QE/g) from the respective TFC of the spiked natural honeys using the quercetin-spiked aqueous honey–methanol solution for blanking.

**Table 5:** Quantitative determination of flavonoids in quercetin-spiked natural and artificial honey using calibration curve 1.

Flavonoid-Spiked Honey	Flavonoid Content (µg QE/g of Honey) (Blanking with Quercetin-Spiked Aqueous Honey–Methanol Solution)	Calculated Natural Flavonoid Content (µg QE/g of Honey)
Red Clover	81.38	12.20
Sainfoin Clover	80.28	11.10
Manuka	100.99	31.81
Jarrah	100.99	31.81
Marri	82.12	12.94
Peppermint	98.61	29.43
Blackbutt	86.15	16.97
Melaleuca	85.78	16.60
Watermelon	100.62	31.44
Bush	101.54	32.36
Artificial honey	69.18	Not applicable

To enhance the respective flavonoid concentration and in doing so lifting the absorbance reading for the natural honeys into the Beer–Lambert range, an alternative assay protocol, using honey extracts, was also investigated. Table 6 presents the TFC of all honey extracts, expressed as micrograms of quercetin per gram of extracted honey, after blanking with the respective aqueous honey extract–methanol solution using the quercetin calibration curve 1.

**Table 6:** TFC of natural honey extracts and quercetin-spiked artificial honey extract using calibration curve 1.

Honey Extract	Flavonoid Content (µg QE/g of Extracted Honey) (Blanking with Aqueous Honey Extract–Methanol Solution)
Red Clover	12.00
Sainfoin Clover	9.40
Manuka	31.23
Jarrah	31.37
Marri	15.80
Peppermint	31.94
Blackbutt	20.01
Melaleuca	18.36
Watermelon	31.19
Bush	32.35
Quercetin-spiked artificial honey	68.52

## 5. Discussion

UV spectrophotometers must be calibrated using a ‘blank’ solution that contains all of the components of the solution to be analysed except for the compound(s) tested for and, in case of a colorimetric assay, the reacting reagent(s) to produce the assay’s typical colour. This blanking step ensures that the recorded absorbance reading only reflects the presence of the analyte without any interference that otherwise would likely result in an overestimation of the assay result. In the colorimetric TFC assay using AlCl_3_ as a reagent, the compound of interest is the Al^3+^ flavonoid coordination complex with its distinct absorbance at around 400 nm. Any inherent honey constituents that naturally also absorb around that wavelength need to be treated as interferences that would lead to an overestimation of flavonoid content and thus their contribution to the absorbance reading needs to be removed in the blanking step. This can be achieved by using an aqueous honey–methanol solution for blanking.

The significance of appropriate blanking can be seen in a comparison of absorbance readings obtained for a range of natural honeys that have been blanked either against a water–methanol solution or a blanking solution consisting of aqueous honey–methanol. In the former case, significant absorbance readings could be detected ranging from 0.365 to 0.778 for the 20% aqueous honey solutions of the 10 honeys of different floral origins that were analysed in this study, whereas the same honey solutions blanked appropriately only recorded negligible absorbance readings ranging from 0.061 to 0.177 (Table 3). These low readings are outside the Beer–Lambert range (0.3–0.8), which ensures linearity between the concentration and absorbance readings, thus should underpin any quantitative UV–Vis spectrophotometric assay. Data obtained for the artificial honey demonstrate that the absorbance seen in natural honeys without appropriate blanking indeed stems from honey’s inherent colour. For the analysis of the artificial honey solution, both blanking approaches resulted in no absorbance reading because this concentrated sugar solution is colourless and not only void of any flavonoids that could complex with Al^3+^ but also does not contain any other constituents that might absorb around 400 nm [25,78].

This finding is interesting as it questions many of the TFC results published for honey. Based on this study, none of the 20% aqueous solutions derived from a range of honeys from different floral sources recorded an absorbance reading within the Beer–Lambert range when appropriately blanked. This does not allow the determination of TFC and thus stands in contrast to many TFC results for honeys reported in the literature, which were derived with inappropriate blanking (e.g., methanol or ethanol) or for which information on the blanking solution used in the assay was not provided (Table 1). The presence of flavonoids has been confirmed in honeys, but their natural flavonoid levels might not produce absorbance readings within the Beer–Lambert range. As previously discussed, being a highly concentrated sugar solution, honey contains only about 3% ‘other’ constituents that comprise simple phenolics, phenolic acids, proteins, amino acids, organic acids, enzymes, and also flavonoids. Thus, it can be assumed that only a very small fraction of the investigated honey sample is accounted for in this assay. This makes the TFC determination using the AlCl_3_ colorimetric method more challenging for honey compared to other natural products.

To confirm that the assay is capable of detecting flavonoids in honey when they are present in sufficient concentration, spiking experiments were carried out. As a model flavonoid, the same amount of quercetin was added to all honey samples, which then, even when blanked against an aqueous honey–methanol solution, resulted in significant absorbance readings within the Beer–Lambert range (Table 4). As the same quantity of quercetin was added to any naturally present flavonoids in the investigated honeys, the final absorbance readings of the spiked honey samples varied, presenting the same trends that had been seen in the honeys prior to spiking; Bush Honey recorded the highest absorbance reading in both studies whereas Sainfoin Clover Honey was the honey with the lowest response, reflecting natural variations in their flavonoid content. The success of the spiking experiment in lifting the absorbance readings into the Beer–Lambert range can also be seen when comparing the absorbance reading of the artificial honey and the spiked artificial honey (Table 3 and Table 4).

To further confirm that the TFC assay for honey is challenged by its naturally low levels of flavonoids, honey extracts were prepared and investigated using the TFC assay. The extraction can be assumed to remove most of the honey’s sugar matrix and thus amplify the concentration of its minor ‘other’ constituents, including its flavonoids. All investigated honey extracts produced absorbance readings within the Beer–Lambert range (0.391 to 0.519) when blanked against an aqueous honey extract–methanol solution, similar to what was seen in the investigated spiked honeys. The trends previously observed for the honeys with and without spiking were also replicated in the honey extracts, with Bush Honey recording the highest and Sainfoin Clover Honey the lowest absorbance reading.

A question arising from these findings is whether the TFC determination for honey using AlCl_3_ is still a feasible method. Based on the generated data, it can be concluded that it is, however, with some modifications to the traditional assay protocol. It is essential that a honey-based blanking solution, for example an aqueous honey–methanol solution as prepared in this study, is used to avoid any overestimation of TFC. It is also recommended that honeys are spiked with a known amount of a model flavonoid such as quercetin to elevate individual absorbance readings into the Beer–Lambert range. Alongside this, an artificial honey also needs to be spiked with the same quantity of the model flavonoid and the TFC of the investigated natural honeys can then be determined by subtracting the absorbance reading of the spiked artificial honey from the respective absorbance reading of the spiked natural honeys. An alternative approach could be to work with honey extracts rather than pure honeys, but in this case, a comparison of the TFC of different honey extracts is only possible if the same extraction protocol is followed, which limits the widespread adoption of this approach. Furthermore, depending on the chosen extraction solvent, not all flavonoids might be accounted for when carrying out the assay with honey extracts.

Quantification of TFC using the difference in absorbance readings of the investigated honeys and also the artificial honey after spiking with the same amount of quercetin was carried out in this study to demonstrate the application of the suggested modification of the assay protocol. The TFC content of the flavonoid-spiked honeys is shown in Table 5 alongside the calculation of their natural flavonoid level, with both values expressed as quercetin equivalents (QE) per gram of the sample, a unit of measurement frequently used to determine TFC. The following equation can be used to calculate the natural total flavonoid content (TFC) of a honey, expressed as Quercetin Equivalents (QE) per gram of the sample:

A_NH_ = A_SNH_ − A_SAH_ (1)

where A_NH_ is the absorbance readings of actual flavonoid levels in natural honey, A_SNH_ is the absorbance readings of flavonoid-spiked natural honey, and A_SAH_ is the absorbance readings of flavonoid-spiked artificial honey.

The natural TFC of the honey is then derived from the linear equation of the calibration curve of quercetin standards obtained after blanking with a quercetin–methanol solution.

Adopting this approach to the quantification of natural total flavonoid levels in the investigated honeys, 12.20, 11.10, 31.81, 31.81, 12.94, 29.43, 16.97, 16.60, 31.44, and 32.36 µg QE/g of honey were determined for Red Clover, Sainfoin Clover, Manuka, Jarrah, Marri, Peppermint, Blackbutt, Melaleuca, Watermelon, and Bush Honey, respectively (Table 5), illustrating the natural variation in TFC in honeys.

As suggested in this study, an alternative to this approach could be the investigation of honey extracts rather than pure honey in the TFC assay while blanking with the respective aqueous honey extract–methanol solution (Table 6) and then to express the TFC of the sample as quercetin equivalent per gram of extracted honey, rather than per gram of honey. Next to being more time-consuming and requiring larger quantities of honey for the analysis due to the incorporated extraction step, it also needs to be acknowledged that in this potential modification of the typical assay protocol, the choice of extraction solvent will influence the determined TFC, so a comparison of the TFC of different honey extracts is only possible when extraction protocols are standardised. In this study, an established extraction method for honey was followed [79] and the trends seen in the TFC of all tested honey that were extracted in this way (Table 6) were comparable to that of the TFC of the honeys themselves (Table 5).

## 6. Conclusions

The findings of this study suggest that the total flavonoid content of honey cannot be reliably determined using the commonly used traditional colorimetric assay protocol. This is not reflective of a general issue with the assay but directly related to the specific chemical composition of honey, a highly concentrated sugar solution with only low natural flavonoid levels. This puts into question the TFC of honeys reported in some previous studies that have followed the traditional assay protocol without appropriate blanking. As a review of recent literature has found that in many studies the adopted blanking solution is not even mentioned, it is possible that reported TFC levels in honey might frequently be overestimated as the recorded absorbances might be influenced by the honey’s inherent colour rather than only its specific flavonoid fraction.

Therefore, in this study, the use of a suitable blanking solution and its impact on the assay was comprehensively explored. Honeys spiked with a known concentration of quercetin, a flavonol commonly used as model flavonoid, served as positive control. An artificial honey, a highly concentrated sugar solution representing the typical sugar and water composition of a natural honey void of its ‘other’ around 3% constituents that gives the honey its usual colour, served as a negative control. Furthermore, the use of organic honey extracts to amplify non-sugar honey constituents was also investigated following the same assay protocol.

It was found that the use of an aqueous honey–methanol solution for blanking is crucial to remove interferences that otherwise lead to an overestimation of the TFC of honey. To lift absorbance readings into the Beer–Lambert range to allow accurate quantification, it is also recommended to spike natural honey and also an artificial honey with a known amount of a model flavonoid such as quercetin. The accurate absorbance reading of the natural honey can then be recorded after subtracting the absorbance reading of the artificial honey. This information can be used to express the sample’s TFC as quercetin equivalent per gram of the sample with reference to a standard curve of the model flavonoid. An alternative, though more laborious approach, is the preparation of honey extracts and an expression of their TFC as quercetin equivalents per gram of extracted honey.

## Figures and Tables

**Table 1:** Variations in analytical methods and standards to determine TFC in honey.

Nectar or Honeydew Floral Source	Bee Species	Blank	Wavelength (nm)	Standard	Reported Range of Results *	Reference
Multifloral	Stingless bee	not specified	420	Rutin	23.7 mg RE/100 g	[26]
Ailanthus (*Ailanthus altissima*), fennel (*Foeniculum* sp.), and raspberry (*Rubus idaeus*)	Honeybee	honey and methanol	415	Quercetin	4.51 to 9.57 mg QE/100 g	[27]
mint (*Mentha* spp.)	Honeybee	honey and methanol	415	Quercetin	6.70 to 12.50 mg QE/100 g	[28]
Juazeiro (*Ziziphus joazeiro* Mart.), malícia (*Mimosa quadrivalvis* L.), jurema branca (*Mimosa arenosa* Willd Poir), and velame branco (*Croton heliotropiifolius* Kunth)	Meliponini	honey and methanol	415	Quercetin	1.90 to 4.40 mg QE/100 g	[29]
Multifloral, combretaceae, vitellaria (*Vitellaria paradoxa*), acacia (*Acacia* spp.), and lannea (*Lannea* spp.)	Honeybee	honey and methanol	415	Quercetin	0.17 to 8.35 mg QE/100 g	[30]
Multifloral	Stingless bee	not specified	415	Quercetin	2.31 to 2.77 mg QE/100 g	[31]
Multifloral	Honeybee	not specified	437	Quercetin	1.64 to 3.01 mg QE/100 g	[32]
Multifloral	Stingless bee	not specified	437	Quercetin	0.05 to 0.07 mg QE/g	[33]
Eucalyptus (*Eucalyptus* sp.), orange blossom (*Citrus sinensis*), acacia (*Acacia* sp.), sucupira (*Pterodon emarginatus*), and multifloral	Honeybee	not specified	415	Quercetin	1.14 to 13.52 mg QE/100 g	[34]
Multifloral	Honeybee	not specified	415	Quercetin	8.06 mg QE/100 g	[35]
Multifloral	Stingless bee	not specified	435	Quercetin	242.57 µg QE/g	[36]
Coconut (*Cocos nucifera* L.), burmese rosewood (*Dalbergia benthami* Prain), red silk cotton tree (*Bombax ceiba* L.), chinese chestnut (*Castanea mollissima* Bl.), and mangrove (Rhizophoraceae)	*Apis cerana*	not specified	405	Rutin	4.02 to 29.22 mg RE/100 g	[37]
Multifloral	Honeybee	honey and methanol	405	Quercetin	17.06 to 58.47 mg QE/g	[38]
Tamarisk (*Tamarix gallica*)	Honeybee	not specified	415	Rutin	63.60 to 83.10 mg RE/100 g	[39]
Rubus (*Rubus* spp.), chestnut (*Castanea sativa*), broom (*Cytisus* spp.), heather (*Erica* spp.), eucalyptus (*Eucalyptus* spp.), clover (*Trifolium* spp.), oak (*Quercus* spp.), and viper’s bugloss (*Echium* spp.)	Honeybee	not specified	425	Quercetin	1.28 to 16.70 mg QE/100 g	[40]
Rapeseed (*Brassica napus*)	Honeybee	methanol	415	Quercetin	9.16 mg QE/100 g	[41]
Arabica coffee (*Coffea arabica*), macrostachys coffee (*Coffea macrostachyus*), niger seed (*Guizotia abyssinica*), ironweed (*Vernonia* spp.), eucalyptus (*Eucalyptus* spp.), and umbrella tree (*Schefflera abyssinica*)	Honeybee	not specified	415	Quercetin	18.60 to 65.00 mg QE/100 g	[42]
Multifloral	Stingless bee	methanol	415	Quercetin	0.20 mg QE/ kg	[43]
Orange (*Citrus sinensis*), eucalyptus (*Eucalyptus* spp.), coffee (*Coffea Arabica*), cipo uva (*Cissus rhombifolia*), quince (*Cydonia oblonga*), monjoleiro (*Acacia polyphylla*), mangrove, and honeydew	Honeybee	not specified	425	Quercetin	0.04 to 0.63 mg QE/100 g	[44]
Multifloral	Honeybee	not specified	430	Quercetin	0.46 to 5.27 mg QE/100 g	[45]
Multifloral	Stingless bee	not specified	425	Quercetin	32.00 to 91.16 mg QE/100 g	[46]
Buckthorn (Rhamnaceae), wild mustard (*Sinapis arvensis*), pea family (Fabaceae), toothpickweed (*Ammi visnaga*), carrot family (Apiaceae), mint family (Lamiaceae), rosemary (*Rosmarinus officinalis*), thyme (*Thymus vulgaris*), and multifloral	Honeybee	not specified	425	Quercetin	5.52 to 20.69 mg QE/100 g	[47]
Multifloral	Honeybee	not specified	415	Quercetin	1.92 to 7.39 mg QE/100 g	[48]
Multifloral	*Apis cerana cerana*, *Apis dorsata*, and *Lepidotrigona flavibasis*	not specified	415	Quercetin	3.39 to 11.67 mg QE/100 g	[49]
Mint (*Mentha* spp.)	Honeybee	not specified	415	Quercetin	6.70 to 11.50 mg QE/100 g	[28]
Multifloral	Honeybee	not specified	420	Rutin	77.97 to 92.87 µg RE/g	[50]
Acacia (*Robinia pseudoacacia*), linden (*Tilia* spp.), rapeseed (*Brassica napus*), sunflower (*Helianthus annuus*), and mint (*Mentha* spp.).	Honeybee	not specified	430	Quercetin	0.44 to 3.97 mg QE/100 g	[51]
Sidr (*Ziziphus* spp.)	Honeybee	not specified	415	Rutin	45.1 to 83.1 mg RE/100 g	[52]
Rapeseed (*Brassica napus*)	Honeybee	not specified	415	Quercetin	77.86 to 425.85 mg QE/kg	[53]
Rhododendron (*Rhododendron ponticum* L.), chestnut (*Castanea sativa* Mill.) lavandula, (*Lavandula Stoechas* L.), astragalus (*Astragalus microcephalus* Willd.), chaste tree (*Vitex agnus castus*), polyfloraland honeydew honeys oak (*Quercus robur* L.), and pine (*Pinus* L.)	Honeybee	not specified	415	Quercetin	0.67 to 6.50 mg QE/100 g	[54]
Sidr (*Ziziphus lotus*) and multifloral	Honeybee	not specified	415	Quercetin	20.44 to 338.56 mg QE/100 g	[13]
Tualang (*Koompassia excelsa*), acacia (*Acacia mangium*), pine (*Pinus* spp.), kelulut, and sumar (*Vachellia tortilis*)	Honeybee, Stingless Bee, and *Apis cerana*	not specified	430	Quercetin	0.03 to 0.11 µg QE/g	[55]
Ling-heather (*Calluna vulgaris* (L.) Hull)	Honeybee	not specified	415	Quercetin	0.71 to 1.69 mg QE/100 g	[56]
Giant goldenrod (*Solidago gigantea*), canada goldenrod (*Solidago canadensis*)	Honeybee	not specified	425	Hyperoside	0.53 to 2.21% hyperoside	[57]
Ulmo (*Eucryphia cordifolia*)	Honeybee	not specified	415	Quercetin	6.09 to 62.44 µmol QE/L	[58]
Azir (*Salvia rosmarinus*), bouchnikha (*Ammi visnaga*), daghmouss (*Euphorbia resinifera*), sadra (*Ziziphus lotus*), latchin (*Citrus sinensis*), multifloral blends, kharob (*Ceratonia siliqua*), khzama (*Lavandula angustifolia*), hamd (*Citrus limon*), chouk (*Silybum marianum*), kebbar (*Capparis spinosa*), bakhenou (*Arbutus unedo*), zandaz (*Bupleurum spinosum*), z’îtra (*Thymus vulgaris*), and zaatar (*Origanum vulgare*)	Honeybee	not specified	420	Quercetin	0.70 to 23.30 mg QE/100 g	[59]
Brazilian monoflorals and manuka	Honeybee	not specified	417	Rutin, Quercetin	0.92 to 7.58 mg RE/100 g, 2.24 to 20.43 mg QE/100 g	[60]
Multifloral	Honeybee	honey and methanol	417	Catechin, Quercetin	3.20 to 7.40 mg CE/100 g, 1.67 to 5.08 mg QE/100 g	[61]
Multifloral	Stingless bee	honey and water	417	Quercetin	1.80 to 2.30 mg QE/g	[62]
Multifloral	Honeybee	not specified	417	Quercetin	5.62 to 6.79 mg QE/g	[63]
Multifloral	Stingless bee	ethanol	417	Quercetin	28 to 300 µg QE/g	[64]
Saharian sidr (*Ziziphus spina*-*christi*)	Honeybee	not specified	415	Quercetin	2.13 mg QE/100 g	[65]
Black locust (*Robinia pseudoacacia*), plectranthus (*Plectranthus rugosus*), and multifloral	Honeybee	not specified	415	Quercetin	1.48 to 4.98 mg QE/100 g	[65]
Multifloral, citrus (*Citrus* spp.), knapweed (*Centaurea hyalolepis*)	honeybee and stingless bee	not specified	425	Rutin	70.62 to 237.25 mg RE/kg	[66]
Arbutus (*Arbutus unedo*), multifloral, dryas (*Dryas octopetala*), asphodelus (*Asphodelus albus*), eucalyptus (*Eucalyptus* spp.), ziziphus (*Ziziphus jujuba*), euphorbia (*Euphorbia* spp.), thymus (*Thymus* vulgaris), citrus (*Citrus* spp.), and quercus (*Quercus* spp.	Honeybee	not specified	415	Quercetin	15.11 to 38.23 mg QE/100 g	[67]
Multifloral		honey and methanol	415	Quercetin	7.97 to 44.99 mg QE/100 g	[68]
Caralluma (*Caralluma europaea*), eucalyptus (*Eucalyptus* spp.), thyme (*Thymus* spp.), orange blossom (*Citrus* x *sinensis*), carob (*Ceratonia siliqua*), jujube (*Ziziphus lotus*), spurge (*Euphorbia* spp.), and multifloral	Honeybee	not specified	430	Rutin	10.43 to 58.28 mg RE/100 g	[69]
Multifloral	Honeybee	not specified	415	Catechin	26.74 to 101.53 mg CE/kg	[70]
Multifloral	Stingless bee	not specified	430	Quercetin	3.74 to 14.85 mg QE/100 g	[71]
Sahrawy (desert plants), zater (*Thymus vulgaris*), flower (various flowers), bardakosh (*Origanum majorana*), black seed (*Nigella sativa*), aashab (wild herbs), and manuka (*Leptospermum scoparium*)	Honeybee	not specified	415	Rutin	20.30 to 32.90 mg RE/100 g	[72]
Multifloral	Honeybee	not specified	415	Quercetin	8.90 to 80.02 mg QE/100 g	[73]
Cactus (Cactaceae), citrus (*Citrus* spp.), gramineae (Poaceae), conifers (Pinophyta), walnut (*Juglans* spp.), and multifloral	Honeybee	not specified	415	Quercetin	1.28 to 7.63 mg QE/100 g	[74]
Multifloral	Honeybee	not specified	415	Quercetin	1.90 to 6.40 mg QE/100 g	[75]
Multifloral	Stingless bee	not specified	415	Quercetin	261.6 to 273.0 mg QE/kg	[76]

* CE—catechin equivalent, QE—quercetin equivalent, RE—rutin equivalent.

**Table 2:** Botanical origin of honey samples.

Honey	Botanical Origin
Red Clover Honey	*Trifolium pratense*
Sainfoin Clover Honey	*Onobrychis viciifolia*
Manuka Honey	*Leptospermum scoparium*
Jarrah Honey	*Eucalyptus marginata*
Marri Honey	*Corymbia calophylla*
Peppermint Honey	*Agonis flexuosa*
Blackbutt Honey	*Eucalyptus patens*
Melaleuca Honey	*Melaleuca alternifolia*
Watermelon Honey	*Citrullus lanatus*
Bush Honey	N.A. (multifloral)

## Data Availability

The original contributions presented in this study are included in the article, further inquiries can be directed to the corresponding author.

## References

[1] Saranraj P., Sivasakthi S., Feliciano G. (2016). Pharmacology of Honey: A review. Adv. Biol. Res..

[2] Hossain M.L., Lim L.Y., Hammer K., Hettiarachchi D., Locher C. (2021). Honey-Based Medicinal Formulations: A Critical Review. Appl. Sci..

[3] El Sohaimy S.A., Masry S.H.D., Shehata M. (2015). Physicochemical characteristics of honey from different origins. Ann. Agric. Sci..

[4] Sultana S., Foster K., Lim L.Y., Hammer K., Locher C. (2022). A Review of the Phytochemistry and Bioactivity of Clover Honeys (*Trifolium* spp.). Foods.

[5] Hossain M.L., Lim L.Y., Hammer K., Hettiarachchi D., Locher C. (2022). A Review of Commonly Used Methodologies for Assessing the Antibacterial Activity of Honey and Honey Products. Antibiotics.

[6] Sultana S., Foster K., Bates T., Hossain M.L., Lim L.Y., Hammer K., Locher C. (2024). Determination of Physicochemical Characteristics, Phytochemical Profile and Antioxidant Activity of Various Clover Honeys. Chem. Biodivers..

[7] Mandal M.D., Mandal S. (2011). Honey: Its medicinal property and antibacterial activity. Asian Pac. J. Trop. Biomed..

[8] Van den Berg A.J., van den Worm E., van Ufford H.C., Halkes S.B., Hoekstra M.J., Beukelman C.J. (2008). An in vitro examination of the antioxidant and anti-inflammatory properties of buckwheat honey. J. Wound Care.

[9] Gambacorta E., Simonetti A., Garrisi N., Intaglietta I., Perna A. (2014). Antioxidant properties and phenolic content of sulla (*Hedysarum* spp.) honeys from Southern Italy. Int. J. Food Sci. Technol..

[10] Alvarez-Suarez J.M., Gasparrini M., Forbes-Hernández T.Y., Mazzoni L., Giampieri F. (2014). The Composition and Biological Activity of Honey: A Focus on Manuka Honey. Foods.

[11] Küçük M., Kolaylı S., Karaoğlu Ş., Ulusoy E., Baltacı C., Candan F. (2007). Biological activities and chemical composition of three honeys of different types from Anatolia. Food Chem..

[12] Al-Kafaween M.A., Alwahsh M., Mohd Hilmi A.B., Abulebdah D.H. (2023). Physicochemical Characteristics and Bioactive Compounds of Different Types of Honey and Their Biological and Therapeutic Properties: A Comprehensive Review. Antibiotics.

[13] Ben Amor S., Mekious S., Allal Benfekih L., Abdellattif M.H., Boussebaa W., Almalki F.A., Ben Hadda T., Kawsar S.M.A. (2022). Phytochemical Characterization and Bioactivity of Different Honey Samples Collected in the Pre-Saharan Region in Algeria. Life.

[14] Capela N., Sarmento A., Simões S., Lopes S., Castro S., Alves da Silva A., Alves J., Dupont Y.L., de Graaf D.C., Sousa J.P. (2023). Exploring the External Environmental Drivers of Honey Bee Colony Development. Diversity.

[15] Donkersley P., Rhodes G., Pickup R.W., Jones K.C., Power E.F., Wright G.A., Kenneth Wilson K. (2017). Nutritional composition of honeybee food stores varies with floral composition. Oecologia.

[16] Panche A.N., Diwan A.D., Chandra S.R. (2016). Flavonoids: An overview. J. Nutr. Sci..

[17] Dias M.C., Pinto D.C.G.A., Silva A.M.S. (2021). Plant Flavonoids: Chemical Characteristics and Biological Activity. Molecules.

[18] Smith W.L., DeWitt D.L., Garavito R.M. (2000). Cyclooxygenases: Structural, cellular, and molecular biology. Annu. Rev. Biochem..

[19] D’Mello P., Gadhwal M., Joshi U., Shetgiri P. (2011). Modeling of COX-2 inhibitory activity of flavonoids. Int. J. Pharm. Sci..

[20] Ullah A., Munir S., Badshah S.L., Khan N., Ghani L., Poulson B.G., Emwas A.-H., Jaremko M. (2020). Important Flavonoids and Their Role as a Therapeutic Agent. Molecules.

[21] Sultana S., Hossain M.L., Sostaric T., Lim L.Y., Foster K.J., Locher C. (2024). Investigating Flavonoids by HPTLC Analysis Using Aluminium Chloride as Derivatization Reagent. Molecules.

[22] Xue S., Cheng J., Ma W., Chen K.L., Liu Y., Li J. (2017). Comparison of Lipoxygenase, Cyclooxygenase, Xanthine Oxidase Inhibitory Effects and Cytotoxic Activities of Selected Flavonoids. Earth Energy Sci..

[23] Aoki T., Akashi T., Ayabe S. (2000). Flavonoids of Leguminous Plants: Structure, Biological Activity, and Biosynthesis. J. Plant Res..

[24] Christ B., Mueller K.H. (1960). On the serial determination of the content of flavonol derivatives in drugs. Arch. Pharm. Ber. Dtsch. Pharm. Ges..

[25] Shraim A.M., Ahmed T.A., Rahman M.M., Hijji Y.M. (2021). Determination of total flavonoid content by aluminum chloride assay: A critical evaluation. Food Sci. Technol..

[26] Mwangi M.W., Wanjau T.W., Omwenga E.O. (2024). Stingless bee honey: Nutritional, physicochemical, phytochemical and antibacterial validation properties against wound bacterial isolates. PLoS ONE.

[27] Martinović L.S., Birkic T.N., Pavlešić T., Planinić A., Gobin I., Ostojić D.M., Pedisić S. (2024). Chemical Characterization of Rare Unifloral Honeys of Ailanthus (*Ailanthus altissima*), Fennel (*Foenicum vulgare*), and Raspberry (*Rubus idaeus*) and their Antimicrobial and Antioxidant Activity. Agric. Res..

[28] Pavlešić T., Poljak S., Ostojić D.M., Lučin I., Reynolds C.A., Kalafatovic D., Martinović L.S. (2022). Mint (*Mentha* spp.) Honey: Analysis of the Phenolic Profile and Antioxidant Activity. Food Technol. Biotechnol..

[29] Sousa J.M., de Souza E.L., Marques G., Meireles B., de Magalhães Cordeiro A.T., Gullón B., Pintado M.M., Magnani M. (2016). Polyphenolic profile and antioxidant and antibacterial activities of monofloral honeys produced by Meliponini in the Brazilian semiarid region. Food Res. Int..

[30] Meda A., Lamien C.E., Romito M., Millogo J., Nacoulma O.G. (2005). Determination of the total phenolic, flavonoid and proline contents in Burkina Fasan honey, as well as their radical scavenging activity. Food Chem..

[31] Romero C.A., Sosa N., Vallejos O.A., Navarro A.S., Yamul D.K., Baldi Coronel B.M. (2024). Physicochemical, microbiological, and sensory properties of stingless bee honey from Argentina. J. Apic. Res..

[32] Albu A., Simeanu C., Pop I.M., Pui A., Tarcău D., Cucu-Man S.M. (2024). Selected Characteristics of Multifloral Honeys from North-Eastern Romania. Agriculture.

[33] Lima A.C.O., Dias E.R., Reis I.M.A., Carneiro K.O., Pinheiro A.M., Nascimento A.S., Silva S.M.P., Carvalho C.A.L., Mendonça A.V.R., Vieira I.J.C. (2024). Ferulic acid as major antioxidant phenolic compound of the *Tetragonisca angustula* honey collected in Vera Cruz—Itaparica Island, Bahia, Brazil. Braz. J. Biol..

[34] Alcoléa M., Junior M.B.S., Oliveira K.A.M., Tussolini L., Leite M.A.G., Honorio-França A.C., França E.L., Pertuzatti P.B. (2024). Bioactive compounds of honey from different regions of Brazil: The effect of simulated gastrointestinal digestion on antioxidant and antimicrobial properties. Food Funct..

[35] Tork I.M., Rashad S., Atwa M.A., Farag S.A. (2023). Comparing the effects of gamma irradiation and thermal processing on unavoidable toxic substances in Egyptian honey. Egypt. J. Chem..

[36] Cheng M.Z.S.Z., Zawawi N., Ooi D.J., Chan K.W., Ismail N., Ishak N.A., Esa N.M. (2023). In Vitro Investigation of Antioxidant and Antidiabetic Properties of Phenolic-Rich Extract from Stingless Bee Honey (*Heterotrigona itama*). Malays. J. Med. Health Sci..

[37] Wu J., Zhao S., Chen X., Jiu Y., Liu J., Gao J., Wang S. (2023). Physicochemical properties, multi-elemental composition, and antioxidant activity of five unifloral honeys from *Apis cerana cerana*. Food Technol. Biotechnol..

[38] Wongsa K., Meemongkolkiat T., Duangphakdee O., Prasongsuk S., Rattanawannee A. (2023). Physicochemical Properties, Phenolic, Flavonoid Contents and Antioxidant Potential of Stingless Bee (*Heterotrigona Itama*) Honey from Thailand. Curr. Res. Nutr. Food Sci..

[39] Hegazi A.G., Guthami F.M.A., Ramadan M.F.A., Gethami A.F.M.A., Craig A.M., El-Seedi H.R., Rodríguez I., Serrano S. (2023). The Bioactive Value of *Tamarix gallica* Honey from Different Geographical Origins. Insects.

[40] Escuredo O., Rodríguez-Flores M.S., Míguez M., Seijo M.C. (2023). Multivariate Statistical Approach for the Discrimination of Honey Samples from Galicia (NW Spain) Using Physicochemical and Pollen Parameters. Foods.

[41] Miłek M., Ciszkowicz E., Sidor E., Hęclik J., Lecka-Szlachta K., Dżugan M. (2023). The Antioxidant, Antibacterial and Anti-Biofilm Properties of Rapeseed Creamed Honey Enriched with Selected Plant Superfoods. Antibiotics.

[42] Tesfaye O. (2023). Evaluating the antioxidant properties of unifloral honey (*Apis mellifera* L.) from Ethiopia. Int. J. Food Sci..

[43] Ferreira A.B., Novaes C.G., de Jesus H.O., Borges J.M.P., Ramos F.S., Pereira A.J., de Oliveira D.M., Aguiar R.M. (2023). Honey of *Tetragonisca angustula* from Southwestern Bahia: Influence of Seasonality on the Physicochemical Profile and Glioma Cell Inhibitory Effect. J. Braz. Chem. Soc..

[44] Archilia M.D., Neto A.A.L., Marcucci M.C., Alonso R.C.B., de Camargo T.C., Camargo R.C., Sawaya A.C.H.F. (2023). Characterization of Brazilian monofloral and polyfloral honey by UHPLC-MS and classic physical-chemical analyses. J. Apic. Res..

[45] Pop I.M., Simeanu D., Cucu-Man S.-M., Pui A., Albu A. (2023). Quality Profile of Several Monofloral Romanian Honeys. Agriculture.

[46] Da Silva L.F.C., Lemos P.V.F., de Souza Santos T., Tavares P.P.L.G., Nascimento R.Q., Almeida L.M.R., de Souza C.O., Druzian J.I. (2023). Storage conditions significantly influence the stability of stingless bee (*Melipona scutellaris*) honey. J. Apic. Res..

[47] Bouddine T., Laaroussi H., Bakour M., Guirrou I., Khallouki F., Mazouz H., Hajjaj H., Hajji L. (2022). Organic Honey from the Middle Atlas of Morocco: Physicochemical Parameters, Antioxidant Properties, Pollen Spectra, and Sugar Profiles. Foods.

[48] Vică M.L., Glevitzky M., Dumitrel G.-A., Bostan R., Matei H.V., Kartalska Y., Popa M. (2022). Qualitative Characterization and Antifungal Activity of Romanian Honey and Propolis. Antibiotics.

[49] Wu J., Han B., Zhao S., Zhong Y., Han W., Gao J., Wang S. (2022). Bioactive characterization of multifloral honeys from *Apis cerana*, *Apis dorsata*, and *Lepidotrigona flavibasis*. Food Res. Int..

[50] Edo G.I., Onoharigho F.O., Akpoghelie P.O., Emakpor O.L., Ozgor E., Akhayere E. (2022). Physicochemical, Phytochemical, Antioxidant, and Inhibition Properties of Key Enzymes Linked to Raw and Regular Honey. Chem. Afr..

[51] Albu A., Radu-Rusu R.-M., Simeanu D., Radu-Rusu C.-G., Pop I.M. (2022). Phenolic and Total Flavonoid Contents and Physicochemical Traits of Romanian Monofloral Honeys. Agriculture.

[52] Hegazi A.G., Al Guthami F.M., Ramadan M.F.A., Al Gethami A.F.M., Craig A.M., Serrano S. (2022). Characterization of Sidr (*Ziziphus* spp.) Honey from Different Geographical Origins. Appl. Sci..

[53] Grabek-Lejko D., Miłek M., Sidor E., Puchalski C., Dżugan M. (2022). Antiviral and Antibacterial Effect of Honey Enriched with *Rubus* spp. as a Functional Food with Enhanced Antioxidant Properties. Molecules.

[54] Yildiz O., Gurkan H., Sahingil D., Atiye Degirmenci A., Kemal M.E., Kolayli S., Hayaloglu A.A. (2022). Floral authentication of some monofloral honeys based on volatile composition and physicochemical parameters. Eur. Food Res. Technol..

[55] Sakika K.A., Saiman M.Z., Zamakshshar N.H., Ahmed I.A., Nasharuddin M.N.A., Hashim N.M. (2022). Analysis of Antioxidant Properties and Volatile Compounds of Honeys from Different Botanical and Geographical Origins. Sains Malays..

[56] Osés S.M., Cantero L., Puertas G., Fernández-Muiño M.A., Sancho M.T. (2022). Antioxidant, antimicrobial and anti-inflammatory activities of ling-heather honey powder obtained by different methods with several carriers. LWT—Food Sci. Technol..

[57] Czigle S., Filep R., Balažová E., Szentgyörgyi H., Balázs V.L., Kocsis M., Purger D., Papp N., Farkas Á. (2022). Antioxidant Capacity Determination of Hungarian-, Slovak-, and Polish-Origin Goldenrod Honeys. Plants.

[58] Velásquez P., Giordano A., Valenzuela L.M., Montenegro G. (2022). Combined antioxidant capacity of Chilean bee hive products using mixture design methodology. LWT—Food Sci. Technol..

[59] Lyoussi B., Bakour M., El-Haskoury R., Imtara H., Hano C. (2022). Characterization of Various Honey Samples from Different Regions of Morocco Using Physicochemical Parameters, Minerals Content, Antioxidant Properties, and Honey-Specific Protein Pattern. J. Food Qual..

[60] Pena Júnior D.S., Almeida C.A., Santos M.C.F., Fonseca P.H.V., Menezes E.V., de Melo Junior A.F., Brandão M.M., de Oliveira D.A., de Souza L.F., Silva J.C. (2022). Antioxidant activities of some monofloral honey types produced across Minas Gerais (Brazil). PLoS ONE.

[61] Yayinie M., Atlabachew M., Tesfaye A., Hilluf W., Reta C., Alemneh T. (2022). Polyphenols, flavonoids, and antioxidant content of honey coupled with chemometric method: Geographical origin classification from Amhara region, Ethiopia. Int. J. Food Prop..

[62] Abdullah H., Ibrahim M., Ahmed I.A., Ramli N., Mhd Jalil A.M., Fatihah N.A.R. (2021). Optimisation of phenolic compounds and antioxidant capacity of Trigona honey and propolis using response surface methodology from fermented food products. Int. Food Res. J..

[63] Yalçın G. (2021). Effects of Thermal Treatment, Ultrasonication, and Sunlight Exposure on Antioxidant Properties of Honey. Turk. J. Pharm. Sci..

[64] Mat Ramlan N.A.F., Md Zin A.S., Safari N.F., Chan K.W., Zawawi N. (2021). Application of Heating on the Antioxidant and Antibacterial Properties of Malaysian and Australian Stingless Bee Honey. Antibiotics.

[65] Ganaie T.A., Masoodi F.A., Rather S.A., Wani S.M. (2021). Physicochemical, antioxidant and FTIR-ATR spectroscopy evaluation of Kashmiri honeys as food quality traceability and Himalayan brand. J. Food Sci. Technol..

[66] Seder N., Rayyan W.A., Dayyih W.A., Al-Natour M.A., Hilmi A.B.M. (2021). Phytochemical Investigation, Comparison and Characterization Study of Malaysian Stingless Bee Honey versus Jordanian Honey by LC-MS/MS. Trop. J. Nat. Prod. Res..

[67] Issaad F.Z., Bouhedjar K., Ikhlef A., Lachlah H., Smain D.H., Boutaghane K., Bensouici C. (2021). Multivariate analysis of physico-chemical, bioactive, microbial and spectral data characterisation of Algerian honey. Food Measure.

[68] Galhardo D., Garcia R.C., Schneider C.R., Braga G.C., Chambó E.D., de França D.L.B., Ströher S.M. (2021). Physicochemical, bioactive properties and antioxidant of *Apis mellifera* L. honey from western Paraná, Southern Brazil. Food Sci. Tech..

[69] Ouradi H., Hanine H., Fauconnier M.L., Kenne T., Rizki H., Ennahli S., Hssaini L. (2021). Determination of physico-biochemical proprieties and composition in volatile constituents by solid phase micro-extraction of honey samples from different botanical and geographical origins in Morocco. J. Apic. Res..

[70] Bayram N.E., Kara H.H., Can A.M., Bozkurt F., Akman P.K., Vardar S.U., Çebi N., Yılmaz M.T., Sağdı O., Dertli E. (2021). Characterization of physicochemical and antioxidant properties of Bayburt honey from the North-east part of Turkey. J. Apic. Res..

[71] Maringgal B., Hashim N., Tawakkal I.S.M.A., Mohamed M.T.M., Hamzah M.H. (2021). Phytochemical content, antioxidant activity and mineral elements of honey produced by four different species of Malaysian stingless bees. Food Res..

[72] Hegazi A.G., Al Guthami F.M., Al Gethami A.F.M., Fouad E.A., Abdou A.M. (2021). Antibacterial activity and characterisation of some Egyptian honey of different floral origin. Bulg. J. Vet. Med..

[73] Amessis-Ouchemoukh N., Maouche N., Otmani A., Terrab A., Madani K., Ouchemoukh S. (2021). Evaluation of Algerian’s Honey in Terms of Quality and Authenticity Based on the Melissopalynology and Physicochemical Analysis and Their Antioxidant Powers. Med. J. Nutr. Metab..

[74] Hernández-Fuentes A.D., Chávez-Borges D., Cenobio-Galindo A.J., Zepeda-Velázquez A.P., Figueira A.C., Jiménez-Alvarado R., Campos-Montiel R.G. (2021). Characterization of total phenol and flavonoid contents, colour, functional properties from honey samples with different floral origins. Int. J. Food Stud..

[75] Kivima E., Tanilas K., Martverk K., Rosenvald S., Timberg L., Laos K. (2021). The Composition, Physicochemical Properties, Antioxidant Activity, and Sensory Properties of Estonian Honeys. Foods.

[76] Chen Y.H., Chuah W.C., Chye F.Y. (2021). Effect of drying on physicochemical and functional properties of stingless bee honey. J. Food Process. Preserv..

[77] Cooper R.A., Molan P.C., Harding K.G. (2002). The sensitivity to honey of Gram-positive cocci of clinical significance isolated from wounds. J. Appl. Microbiol..

[78] Liu H., Song Y., Zhang X. (2017). Determination of Total Flavonoids in Leek by AlCl_3_ Colorimetric Assay. Chem. Eng. Trans..

[79] Hossain M.L., Lim L.Y., Hammer K., Hettiarachchi D., Locher C. (2023). Design, Preparation, and Physicochemical Characterisation of Alginate-Based Honey-Loaded Topical Formulations. Pharmaceutics.

